# Risk and protective factors for nonsuicidal self-injury among adolescents: a latent profile analysis

**DOI:** 10.3389/fpsyt.2025.1713461

**Published:** 2025-12-04

**Authors:** Yunfeng He, Yaxin Kong, Tianyuan Ji, Ruoge Tao, Liping Ge, Feng Yuan

**Affiliations:** 1 Liaoning Key Laboratory of Psychological Testing and Behavior Analysis, Liaoning University, Shenyang, China; 2 Luoyang Dongsheng No.1 Primary School, Luoyang, China; 3 Faculty of Psychology, Tianjin Normal University, Tianjin, China; 4 The Gifted Division of Northeast Yucai School, Northeast Yucai School, Shenyang, China; 5 School of Discipline Inspection and Supervision, Liaoning University, Shenyang, China

**Keywords:** non-suicidal self-injury, latent profile analysis, adolescents, comprehensive assessment, influencing factors

## Abstract

**Introduction:**

Despite the longstanding interest in the factors influencing nonsuicidal self-injury (NSSI), less attention has been paid to how risk and protective factors interact to influence adolescents' NSSI behaviors. NSSI is a serious psychological crisis with complex etiology that is usually not triggered by a single chance event. Therefore, assessing NSSI based on risk factors alone is inherently inaccurate. In order to provide a more accurate and comprehensive risk assessment framework, we explored the specific patterns of combinations of risk and protective factors within intra-individual and environmental of adolescents involved in NSSI.

**Methods:**

1091 participants were evaluated on six indicators: depression, emotion regulation, regulatory emotional self-efficacy, resilience, family functioning, and social support.

**Results:**

The results showed three latent profiles: low risk-high protection (30.62%), medium risk-medium protection (58.20%), and high risk-low protection (11.18%). Compared to low risk-high protection, adolescents in medium risk-medium protection (OR = 2.49) and high risk-low protection (OR = 11.46) were significantly associated with increased odds of experiencing NSSI.

**Discussion:**

The findings suggest that we should focus our prevention efforts on a group of adolescents with high-risk-low-protective characteristics to effectively reduce the incidence of NSSI behaviors by enhancing their protective factors or reducing their risk factors.

## Introduction

Non-suicidal self-injury (NSSI) is defined as direct, intentional damage to one’s own body tissues with no suicidal intent (1). A meta-analysis showed that the lifetime prevalence of NSSI among a worldwide nonclinical sample of adolescents was 22.0% (2). In China, a nationwide survey revealed that approximately 29% engaged in at least one episode of NSSI in the past year among 15,623 rural adolescents (3). Given the high risk and prevalence of NSSI, psychological researchers have conducted comprehensive and profound theoretical and empirical research on this complex phenomenon.

NSSI is a serious psychological crisis (4) with compounding causal factors that are typically not triggered by a single chance event (5). These risk factors interact and combine in two patterns: cumulative effect and clustering effect (6). The cumulative effect emphasizes that the accumulation of risk factors increases the probability of psychological crisis. In adolescents, the prevalence of NSSI behaviors has been observed to rise significantly, attributable to the cumulative impact of various risk factors (7). However, we still lack sufficient knowledge on how protective factors moderate self-injurious behavior in the context of risk factors. Rogers (8) emphasized that “any comprehensive examination of risk factors must also consider protective factors” and that “assessments based solely on risk are inherently inaccurate.” This perspective has subsequently been widely endorsed by other scholars (9, 10). Therefore, attaining a more comprehensive understanding of how various risk and protective factors are associated with NSSI is crucial for assessing the risk of NSSI and reducing such behaviors among adolescents in China.

An integrated theoretical model of NSSI incorporates considerations from biological, psychological, and social domains (1). It indicates that some individuals’ internal or interpersonal vulnerabilities may make them more likely to respond to stressful events with emotional or social dysfunction, which may lead to the use of NSSI or other extreme behaviors to regulate feelings. These vulnerabilities are believed to be shaped by distal factors such as family, interpersonal relationships, and other environment variables (1). Therefore, based on an integrative model, the present study adopted an integrative perspective to examine how the combination and interaction of intra-individual factors (e.g., emotion regulation, regulatory emotional self-efficacy, depression, and resilience) and distal factors (e.g., family functioning and social support).

Various theoretical models and empirical studies suggest that NSSI may result from deficits in emotion regulation. For example, the Experiential Avoidance Model (EAM) suggests that NSSI is primarily maintained through negative reinforcement in the form of avoidance or escape from undesired emotional experiences (11). Regarding previous studies, individuals who engage in NSSI demonstrated higher levels of self-reported emotion dysregulation (12, 13) and experienced greater negative emotions (14, 15). The assessment of emotion regulation difficulties has proven to be an effective tool in differentiating 64% of individuals engaging in non-suicidal self-injury (NSSI) from those who do not, achieving a remarkable accuracy rate of 80%. (16). NSSI has been linked to impaired emotion regulation function and a trait of emotional dysregulation (17). Individuals reported that engaging in NSSI was for the most prominent purpose of relief from negative emotions (18, 19). Together, these findings indicate that relieving emotions could serve as a pivotal function in the engagement of NSSI. Therefore, three variables related to emotions were selected in this study: emotion regulation strategies, regulatory emotional self-efficacy and depression.

Gross (20) proposed two emotion regulation strategies: cognitive reappraisal and expressive suppression. Cognitive reappraisal, which typically correlates with psychological well-being, involves individuals reinterpreting situations as a way of regulating their emotions (21). However, chronic reliance on externally expressing suppressed emotions may increase the physiological stress response (21). Previous studies have revealed a positive association between NSSI and expression suppression, along with a negative correlation between NSSI and cognitive reappraisal (22, 23). Adolescents who engaged in NSSI reported higher expression inhibition scores and lower cognitive reappraisal scores (24).

The cognitive-emotional model of NSSI proposes that self-efficacy has an important role in the initiation and maintenance of NSSI (25). Bandura (26) made it clear that task-specific self-efficacy is a significant predictor of behavior. Therefore, we included regulatory emotional self-efficacy in our analyses. It plays an important role in the process of emotion regulation, referring to an individual’s level of confidence and perceived ability to regulate emotions (27). High regulatory emotional self-efficacy acts as a protective factor against NSSI in adolescents (28), moderating the relationship between NSSI and risk factors (29). When self-efficacy for managing frustration/pain is high, adolescents are more likely to use positive coping strategies to relieve negative emotions and are less inclined to use NSSI (30, 31).

As NSSI is frequently used as an emotion regulation strategy, adolescents experiencing depression may resort to NSSI as a coping mechanism. Previous research has indicated a positive association between depression and NSSI (32, 33), with NSSI adolescents reporting higher levels of depression (34). A meta-analysis revealed that adaptive emotion regulation strategies were negatively correlated with depression, whereas maladaptive emotion regulation strategies showed a positive association (35). Effective emotion regulation strategies can diminish feelings of depression, consequently reducing NSSI behaviors (36).

Resilience is closely related to positive emotions and represents an individual’s capacity to develop positive adaptive skills and maintain or regain mental health when confronted with adversity (37). The relationship between resilience and emotion regulation is both evident and distinct (38). Cognitive reappraisal leads to adaptive emotional responses in the face of adversity, thus contributing to increased resilience (39). Individuals with high levels of resilience are more adept at regulating their emotions in challenging situations and are tend to recover from negative emotions more swiftly (40). Distal risk factors (e.g., abuse) may lead to profoundly aversive emotional responses to stress, which may consequently trigger NSSI (41). However, resilience can help individuals maintain a positive mental state in the face of a stressful event (42), leading to positive coping styles rather than NSSI. Moreover, positive emotions can increase resilience levels to the extent of being able to cope with stress effectively (43).

In addition to individual psychological factors, the importance of the environment in individual development and mental health cannot be neglected. The relational developmental systems theory assumes that the interaction between an individual and their environment stimulates the individual’s development (44). The interpersonal/systemic model suggests that an individual’s NSSI behavior is the result of environmental or family dysfunction (45, 46). The family, as a key environment for development, plays a crucial role in shaping and maintaining mental health (47). Persistent and immediate family dysfunction poses a particularly severe threat to adolescents, significantly increasing the risk of NSSI among them (48, 49). Poor family functioning, characterized by inadequate communication and interaction among family members, coupled with a deficiency in collaborative problem-solving and stress-coping abilities, has been associated with various negative outcomes (50). Consequently, adolescents in such environments may be more inclined to adopt unhealthy coping mechanisms, such as NSSI (51). Adaptive emotion regulation moderates the relationship between family functioning and recovery from NSSI (52). Existing within a dysfunctional family environment significantly also increases adolescents’ risk of experiencing depressive symptoms, which in turn may contribute to a greater tendency to resort to NSSI as a way of coping with negative emotions (51).

Apart from the family, other crucial sources of social support include school, friends, and the community. Social support played a protective role against NSSI, helping to moderate or alleviate the negative effect of risk factors on adolescents (53–55). Moreover, it played a mediating role between emotion regulation ability and NSSI (56), while the perceived ability to regulate emotion functions as a mediating factor in the relationship between social support and NSSI (57). Both family functioning and social support jointly contribute to preventing self-injurious behavior (58).

Most previous studies have been based on variable-centered approaches to analyze the relationship between one or a few risk factors and NSSI. But in reality, NSSI does not occur as a result of a single factor, but rather as a result of the synergistic interaction of multiple factors. In addition, the use of a single indicator for screening priority populations may directly contribute to the problem of high false-positive rates. Some researchers have also applied the methods of person-oriented analysis to identify risks related to NSSI, such as Latent Class Analysis (LCA) and Latent Profile Analysis (LPA). The variable-centered approaches focus on exploring commonalities among research participants, while the person-oriented approaches initially identify potential heterogeneity among participants and then explore commonalities after dividing them into subgroups based on their response patterns, thereby enhancing the precision of the analysis (59) and more in line with reality (60). However, previous research on NSSI from a person-oriented analytic perspective has mostly focused on risk factors for NSSI (61), characteristics of NSSI itself (e.g., frequency, modality, functioning, motivation, etc) (62, 63), or NSSI and other internalizing symptoms (e.g., depression, borderline personality disorder, dysregulated eating, etc) (64–66). Risk and protective factors are less integrated and therefore do not better reflect the reality of the situation. Consequently, research on this crucial topic is necessary to more accurately guide the prevention of NSSI in adolescents.

In summary, we investigated the pattern of combinations of individual psychological and environmental risk (e.g depression, expressive suppression, family functioning) and protective factors (e.g. cognitive reappraisal, regulatory emotional self-efficacy, resilience, social support) and the association between this pattern of combinations and NSSI by LPA.

Non-suicidal self-injury (NSSI) is defined as direct, intentional damage to one’s own body tissues with no suicidal intent (1). A meta-analysis showed that the lifetime prevalence of NSSI among a worldwide nonclinical sample of adolescents was 22.0% (2). In China, a nationwide survey revealed that approximately 29% engaged in at least one episode of NSSI in the past year among 15,623 rural adolescents (3). Given the high risk and prevalence of NSSI, psychological researchers have conducted comprehensive and profound theoretical and empirical research on this complex phenomenon.

NSSI is a serious psychological crisis (4) with compounding causal factors that are typically not triggered by a single chance event (5). These risk factors interact and combine in two patterns: cumulative effect and clustering effect (6). The cumulative effect emphasizes that the accumulation of risk factors increases the probability of psychological crisis. In adolescents, the prevalence of NSSI behaviors has been observed to rise significantly, attributable to the cumulative impact of various risk factors (7). However, we still lack sufficient knowledge on how protective factors moderate self-injurious behavior in the context of risk factors. Rogers (8) emphasized that “any comprehensive examination of risk factors must also consider protective factors” and that “assessments based solely on risk are inherently inaccurate.” This perspective has subsequently been widely endorsed by other scholars (9, 10). Therefore, attaining a more comprehensive understanding of how various risk and protective factors are associated with NSSI is crucial for assessing the risk of NSSI and reducing such behaviors among adolescents in China.

An integrated theoretical model of NSSI incorporates considerations from biological, psychological, and social domains (1). It indicates that some individuals’ internal or interpersonal vulnerabilities may make them more likely to respond to stressful events with emotional or social dysfunction, which may lead to the use of NSSI or other extreme behaviors to regulate feelings. These vulnerabilities are believed to be shaped by distal factors such as family, interpersonal relationships, and other environment variables (1). Therefore, based on an integrative model, the present study adopted an integrative perspective to examine how the combination and interaction of intra-individual factors (e.g., emotion regulation, regulatory emotional self-efficacy, depression, and resilience) and distal factors (e.g., family functioning and social support).

Various theoretical models and empirical studies suggest that NSSI may result from deficits in emotion regulation. For example, the Experiential Avoidance Model (EAM) suggests that NSSI is primarily maintained through negative reinforcement in the form of avoidance or escape from undesired emotional experiences (11). Regarding previous studies, individuals who engage in NSSI demonstrated higher levels of self-reported emotion dysregulation (12, 13) and experienced greater negative emotions (Bresin, 2014; 15). The assessment of emotion regulation difficulties has proven to be an effective tool in differentiating 64% of individuals engaging in non-suicidal self-injury (NSSI) from those who do not, achieving a remarkable accuracy rate of 80%. (16). NSSI has been linked to impaired emotion regulation function and a trait of emotional dysregulation (17). Individuals reported that engaging in NSSI was for the most prominent purpose of relief from negative emotions (18, 19). Together, these findings indicate that relieving emotions could serve as a pivotal function in the engagement of NSSI. Therefore, three variables related to emotions were selected in this study: emotion regulation strategies, regulatory emotional self-efficacy and depression.

Gross (20) proposed two emotion regulation strategies: cognitive reappraisal and expressive suppression. Cognitive reappraisal, which typically correlates with psychological well-being, involves individuals reinterpreting situations as a way of regulating their emotions (21). However, chronic reliance on externally expressing suppressed emotions may increase the physiological stress response (21). Previous studies have revealed a positive association between NSSI and expression suppression, along with a negative correlation between NSSI and cognitive reappraisal (22, 23). Adolescents who engaged in NSSI reported higher expression inhibition scores and lower cognitive reappraisal scores (24).

The cognitive-emotional model of NSSI proposes that self-efficacy has an important role in the initiation and maintenance of NSSI (25). Bandura (26) made it clear that task-specific self-efficacy is a significant predictor of behavior. Therefore, we included regulatory emotional self-efficacy in our analyses. It plays an important role in the process of emotion regulation, referring to an individual’s level of confidence and perceived ability to regulate emotions (27). High regulatory emotional self-efficacy acts as a protective factor against NSSI in adolescents (28), moderating the relationship between NSSI and risk factors (29). When self-efficacy for managing frustration/pain is high, adolescents are more likely to use positive coping strategies to relieve negative emotions and are less inclined to use NSSI (30, 31).

As NSSI is frequently used as an emotion regulation strategy, adolescents experiencing depression may resort to NSSI as a coping mechanism. Previous research has indicated a positive association between depression and NSSI (32, 33), with NSSI adolescents reporting higher levels of depression (34). A meta-analysis revealed that adaptive emotion regulation strategies were negatively correlated with depression, whereas maladaptive emotion regulation strategies showed a positive association (35). Effective emotion regulation strategies can diminish feelings of depression, consequently reducing NSSI behaviors (36).

Resilience is closely related to positive emotions and represents an individual’s capacity to develop positive adaptive skills and maintain or regain mental health when confronted with adversity (37). The relationship between resilience and emotion regulation is both evident and distinct (38). Cognitive reappraisal leads to adaptive emotional responses in the face of adversity, thus contributing to increased resilience (39). Individuals with high levels of resilience are more adept at regulating their emotions in challenging situations and are tend to recover from negative emotions more swiftly (40). Distal risk factors (e.g., abuse) may lead to profoundly aversive emotional responses to stress, which may consequently trigger NSSI (41). However, resilience can help individuals maintain a positive mental state in the face of a stressful event (42), leading to positive coping styles rather than NSSI. Moreover, positive emotions can increase resilience levels to the extent of being able to cope with stress effectively (43).

In addition to individual psychological factors, the importance of the environment in individual development and mental health cannot be neglected. The relational developmental systems theory assumes that the interaction between an individual and their environment stimulates the individual’s development (44). The interpersonal/systemic model suggests that an individual’s NSSI behavior is the result of environmental or family dysfunction (45, 46). The family, as a key environment for development, plays a crucial role in shaping and maintaining mental health (47). Persistent and immediate family dysfunction poses a particularly severe threat to adolescents, significantly increasing the risk of NSSI among them (48, 49). Poor family functioning, characterized by inadequate communication and interaction among family members, coupled with a deficiency in collaborative problem-solving and stress-coping abilities, has been associated with various negative outcomes (50). Consequently, adolescents in such environments may be more inclined to adopt unhealthy coping mechanisms, such as NSSI (51). Adaptive emotion regulation moderates the relationship between family functioning and recovery from NSSI (52). Existing within a dysfunctional family environment significantly also increases adolescents’ risk of experiencing depressive symptoms, which in turn may contribute to a greater tendency to resort to NSSI as a way of coping with negative emotions (51).

Apart from the family, other crucial sources of social support include school, friends, and the community. Social support played a protective role against NSSI, helping to moderate or alleviate the negative effect of risk factors on adolescents (53–55). Moreover, it played a mediating role between emotion regulation ability and NSSI (56), while the perceived ability to regulate emotion functions as a mediating factor in the relationship between social support and NSSI (57). Both family functioning and social support jointly contribute to preventing self-injurious behavior (58).

Most previous studies have been based on variable-centered approaches to analyze the relationship between one or a few risk factors and NSSI. But in reality, NSSI does not occur as a result of a single factor, but rather as a result of the synergistic interaction of multiple factors. In addition, the use of a single indicator for screening priority populations may directly contribute to the problem of high false-positive rates. Some researchers have also applied the methods of person-oriented analysis to identify risks related to NSSI, such as Latent Class Analysis (LCA) and Latent Profile Analysis (LPA). The variable-centered approaches focus on exploring commonalities among research participants, while the person-oriented approaches initially identify potential heterogeneity among participants and then explore commonalities after dividing them into subgroups based on their response patterns, thereby enhancing the precision of the analysis (59) and more in line with reality (60). However, previous research on NSSI from a person-oriented analytic perspective has mostly focused on risk factors for NSSI (61), characteristics of NSSI itself (e.g., frequency, modality, functioning, motivation, etc) (62, 63), or NSSI and other internalizing symptoms (e.g., depression, borderline personality disorder, dysregulated eating, etc) (64–66). Risk and protective factors are less integrated and therefore do not better reflect the reality of the situation. Consequently, research on this crucial topic is necessary to more accurately guide the prevention of NSSI in adolescents.

In summary, we investigated the pattern of combinations of individual psychological and environmental risk (e.g depression, expressive suppression, family functioning) and protective factors (e.g. cognitive reappraisal, regulatory emotional self-efficacy, resilience, social support) and the association between this pattern of combinations and NSSI by LPA.

## Methods

### Participants

A total of 1234 first-year university students participated in the questionnaire survey. 143 questionnaires were excluded due to omissions and missing key variables (i.e., NSSI). The final sample consisted of 1091 participants (*M*_age_ = 18.75 years, *SD* = 0.80; 81.6% females). 196 (18.0%) adolescents reported engaging at least one NSSI behavior. Before participating, informed consent was obtained from all participants through the provision of consent forms. Participation was voluntary, informed, and anonymous.

### Measures

#### Demographic date

Demographic characteristics included age, gender, only/non-only child in the family, and monthly family income.

#### Family assessment device

Family function was assessed using the Family Assessment Device (FAD, 50, 67), which consists of 60 items and is rated on a 4-point Likert scale, ranging from 1 (“strongly agree”) to 4 (“strongly disagree”). The general functioning sub-scale was used in this study to rate the functioning of the family in general, with 12 items. Higher mean scores indicate poorer family general functioning. The Cronbach’s alpha for the general functioning scale in the present study was 0.86.

#### Beck depression inventory

The Beck Depression Scale developed by Beck (1977) and revised by Chen et al. (68). It has been widely used to measure the severity of depression. This scale consists of 21 items and participants answer the items on a four-point Likert scale from 0 (“I do not feel sad”) to 3 (“I am so sad or unhappy that I can’t stand it”), with higher scores indicating more severe depression. The Cronbach’s alpha in the present study was 0.93.

#### Emotion regulation questionnaire

The Emotion Regulation Strategies Questionnaire (ERQ, 21, 69) is a 10-item self-report questionnaire that measures two strategies of emotional regulation: expresses suppression and cognitive reappraisal. The items are rated on a 7-point Likert scale, ranging from 1 (“strongly disagree”) to 7 (“strongly agree”). Higher scores on each dimension suggest a higher level of emotional regulation. In the study sample, Cronbach’s alpha for expressive suppression and cognitive reappraisal were 0.73 and 0.83, respectively.

#### Regulatory emotional self-efficacy scale

The Emotion Regulation Self-Efficacy Scale (27, 70) is a 12-item scale. Participants rate on a 5-point Likert scale, ranging from 1 (“not well at all”) to 5 (“very well”). Higher total scores indicate greater individual self-efficacy in emotion regulation. The Cronbach’s alpha for the total scale was 0.80 in the present study.

#### Connor-Davidson Resilience Scale

The Connor-Davidson Resilience Scale, originally developed by Connor and Davidson (71) and subsequently revised by Yu and Zhang (72), was utilized in this study. The Scale, utilized to measure tenacity, strength, and optimism, comprises 25 items and is scored on a 5-point Likert scale ranging from 0 (“not true at all”) to 4 (“true nearly all of the time”). Higher total scores indicate higher psychological resilience. The Cronbach’s alpha for the total scale was 0.94 in this study.

#### Social support rating scale

The Social Support Rating (SSRE, 73) contains 10 items that assess three aspects: subjective support, objective support, and availability of support. The Cronbach’s alpha was 0.78 in the present study.

#### Adolescents self-harm scale

The Adolescents Self-Harm Scale (ASHS) was developed by Zheng (74) and revised by Feng (75) with 19 items. “Number of occurrences” is assessed using a 4-point Likert scale ranging from 0 (“none”) to 3 (“more than 5 times (including 5 times)”) and “degree of body harm” refers to the objective presence of harm and is rated on a 5-point scale from 0 (“none”) to 4 (“very severe”). Lastly, a cumulative total score is obtained for all the items (self-injurious behavior = number of occurrences * degree of harm to the body). Higher total scores indicate a more severe pattern of NSSI. In this study, the scores of NSSI were divided into a binary variable, with “none” meaning that the cumulative total score is 0, and “yes” meaning that the cumulative score is greater than or equal to 1. The Cronbach’s alpha for this study sample was 0.96.

### Statistical analysis

LPA was performed using Mplus 8.0 to explore patterns of combinations of risk and protective factors in adolescents. Before conducting the LPA, standardized scores were converted for each class indicator. We started with a zero model (i.e., an initial model with an initial number of latent profiles of one) and then gradually increased the number of latent profiles for parameter estimation of each model. We calculated several statistics to select the best model: Log Likelihood (LL), Akaike Information Criterion (AIC), Bayesian Information Criterion (BIC), the sample-size-adjusted BIC (ABIC), Lo-Mendell-Rubin Likelihood Adjusted Ratio Test (LMR), Bootstrap Likelihood Ratio Test (BLRT), and Entropy. The LL, AIC, BIC, and ABIC values serve for model comparison, with lower values indicating better model fit. The values of LMR and BLRT reach the significance level (*p < 0*.05), which indicates that the model of the k profile is better than the k-1 profile model. Entropy indicates the classification accuracy of the model (range 0-1), with a score closer to 1 indicating a better model fit (generally required to Entropy ≥ 0.8) (76). Subsequently, logistic regression analysis was then performed using SPSS 22.0 to analyze the relationship between the different latent profiles and NSSI.

## Results

### Description analysis

The results of the demographic information of the participants are shown in **Table 1**. An independent samples t-test was used to compare whether the participants differed on demographic variables for the main variables, as shown in **Table 2**. The results found that there was a significant difference in resilience (*t* = -1.44, *p* < 0.01) and social support (*t* = 1.66, *p* < 0.01) by gender. Boys had higher resilience scores and girls had higher social support scores. NSSI (*t* = 1.97, *p* < 0.01), resilience (*t* = 1.16, *p* < 0.05) and social support (*t* = -3.90, *p* < 0.01) differed significantly on only/non-only child. Only children had higher scores for NSSI and resilience, but lower scores for social support.

**Table 1:** Description of participants (*n* = 1091).

Variable	*N* (%)/*M* ± *SD*	Variable	*N* (%)
*Age (years)*	18.75 ± 0.80	*Monthly family income*	
*Gender*		Less than 2,999	117 (10.7%)
Boys	201 (18.4%)	3,000-5,999	371 (34.0%)
Girls	890 (81.6%)	6,000-8,999	306 (28.1%)
*Only/non-only child*		9,000-11,999	170 (15.6%)
Yes	496 (45.4%)	More than 12,000	126 (11.6%)
No	596 (54.6%)	*Nonsuicidal self-injury*	
		Yes	196 (18.0%)
		No	895 (82.0%)

**Table 2:** Differences in demographic variables of the main variables.

	Nonsuicidal self-injury		Depression		Expresses suppression		Family functioning		Cognitive reappraisal		Regulatory emotional self-efficacy		Resilience		Social support	
	*M ± SD*	*t*	*M ± SD*	*t*	*M ± SD*	*t*	*M ± SD*	*t*	*M ± SD*	*t*	*M ± SD*	*t*	*M ± SD*	*t*	*M ± SD*	*t*
*Gender*																
Boys (*n* = 201)	0.92 ± 2.41	-0.27	5.72 ± 8.36	-0.93	16.03 ± 4.60	-4.00	23.48 ± 5.51	-1.30	28.42 ± 5.45	0.32	42.17 ± 6.27	-2.81	61.65 ± 15.55	-1.44^**^	36.10 ± 5.89	1.66^**^
Girls (*n* = 890)	0.86 ± 2.97		5.17 ± 7.42		14.7 ± 04.20		22.95 ± 5.14		28.55 ± 5.23		40.87 ± 5.83		60.12 ± 13.11		36.78 ± 5.08	
*Only/non-only child*																
Yes (*n* = 495)	1.06 ± 3.03	1.97^**^	5.29 ± 7.76	0.06	15.06 ± 4.37	0.79	22.50 ± 5.36	-3.20	28.55 ± 5.48	0.16	41.44 ± 5.92	1.69	60.92 ± 14.40	1.16^*^	35.98 ± 5.56	-3.90^**^
No (*n* = 596)	0.71 ± 2.73		5.26 ± 7.48		14.85 ± 4.25		23.51 ± 5.05		28.50 ± 5.10		40.83 ± 5.94		59.97 ± 12.89		37.22 ± 4.90	

^*^p < 0.05; ^**^p < 0.01; ^***^p < 0.001; the same as below.

### Correlation analyses

The results of the correlation analyses are shown in **Table 3**. NSSI was significantly positively correlated with family functioning (*r* = 0.18, *p* < 0.001), expresses suppression (*r* = 0.10, *p* < 0.01), and depression (*r* = 0.37, *p* < 0.001). It was significantly negatively correlated with cognitive reappraisal (*r* = -0.13, *p* < 0.001), regulatory emotional self-efficacy (*r* = -0.14, *p* < 0.001), resilience (*r* = -0.11, *p* < 0.001), and social support (*r* = -0.15, *p* < 0.001).

**Table 3:** Correlation analysis between variables.

Variable	*M* ± *SD*	1	2	3	4	5	6	7
1. depression	5.27 ± 7.60							
2. expresses suppression	14.94 ± 4.30	0.16^***^						
3. family functioning	23.05 ± 5.21	0.41^***^	0.22^***^					
4. cognitive reappraisal	28.52 ± 5.27	-0.25^***^	0.03	-0.31^***^				
5. regulatory emotional self-efficacy	41.11 ± 5.93	-0.36^***^	-0.13^***^	-0.34^***^	0.44^***^			
6. social support	36.65 ± 5.24	-0.41^***^	-0.23^***^	-0.44^***^	0.26^***^	0.39^***^		
7. resilience	60.40 ± 13.60	-0.44^***^	-0.08^**^	-0.43^***^	0.44^***^	0.56^***^	0.51^***^	
8. NSSI	0.87 ± 2.88	0.37^***^	0.10^**^	0.18^***^	-0.13^***^	-0.14^***^	-0.15^***^	-0.11^***^

**p < 0.01; ***p < 0.001.

### Latent profile analysis

**Figure 1:**
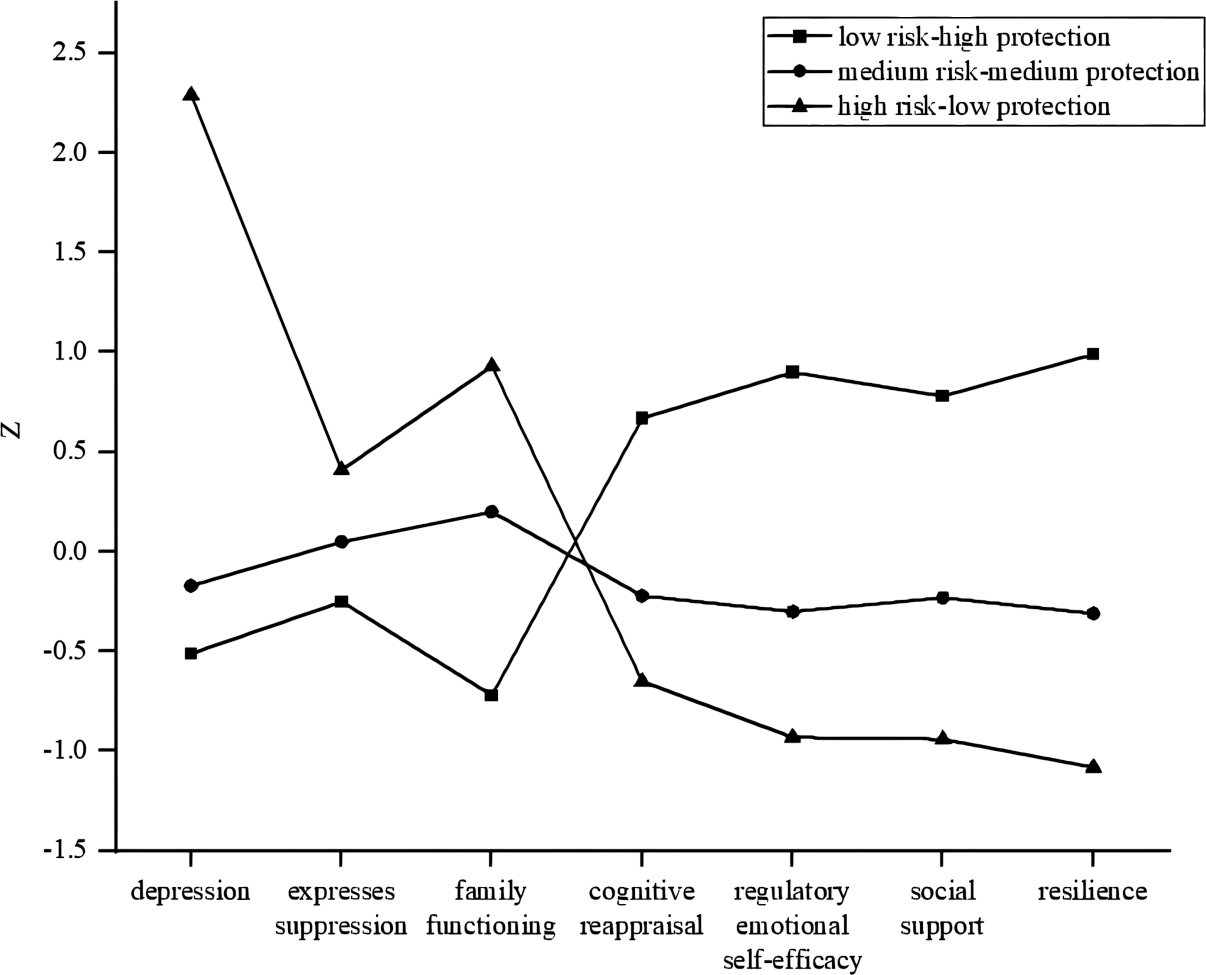
Latent profiles of risk and protective factors.

Models ranging from one to four profile solutions were evaluated using seven class indicators, and the resulting fit indices are presented in **Table 4**. The LL, AIC, BIC and ABIC indicators all decreased gradually with the increase of the number of profiles, and the Entropy values were all above 0.8. However, the four-profile model LMR did not reach the level of significance, indicating that the three-profile model fits better than the four-profile model. In addition, numbers in each category of the three-profile model were higher than 5%, and considering the simplicity of the model, it was the best-fitting model (see **Figure 1**). Factors affecting NSSI in adolescents can be divided into three profiles: (1) low risk-high protection (*n* = 334, 30.62%), (2) medium risk-medium protection (*n* = 635, 58.20%) and (3) high risk-low protection (*n* = 112, 11.18%).

**Table 4:** Fit indices for models with one-to-four profiles.

Modal	LL	AIC	BIC	ABIC	pLMR	pBLRT	Entropy	Group Size
1	-10832.93	21693.87	21763.80	21719.33				1091(100%)
2	-10247.41	20538.83	20648.71	20578.83	<0.001	<0.001	0.92	909(83.32%) 182(16.68%)
3	**-9915.97**	**19891.93**	**20041.78**	**19946.49**	**<0.01**	**<0.001**	**0.80**	**334(30.62%)** **635(58.20%)** **122(11.18%)**
4	-9750.57	19577.13	19766.94	19646.24	0.5221	<0.001	0.84	152(13.93%) 287(26.31%) 623(57.10%) 29(2.66%)

LL, Log Likelihood; AIC, Akaike Information Criterion, BIC, Bayesian Information Criterion; ABIC, the sample-size-adjusted BIC; LMR, Lo-Mendell-Rubin Likelihood Adjusted Ratio Test; BLRT, Bootstrap Likelihood Ratio Test. Bolded values indicate that the three-class model represents the optimal fit.

### Logistic regression analysis

Participants were categorized into two groups based on their NSSI scores: an NSSI group with scores ≥ 1, and a non-NSSI group with scores of 0. To examine the relationship between distinct latent profiles and NSSI, a binomial logistic regression analysis was conducted, incorporating gender and being an only/non-only child as control variables. The results are shown in **Table 5**. Only children were nearly twice as likely to engage in NSSI compared to non-only children. (*p* < 0.01). Adolescents with medium risk-medium protection were over twice as likely, and those with high risk-low protection were more than 11 times as likely, to engage in NSSI compared to adolescents with low risk-high protection. (all *p* < 0.001).

**Table 5:** Comparison of different profiles of NSSI.

Variable	NSSI (yes vs no)		
	B	Wald	OR (95%CI)
Gender			
Boys	0		
Girls	-0.38	3.38	0.69 [0.46, 1.03]
Only/non-only child			
Yes	0.47^**^	7.81	1.60 [1.15, 2.23]
No	0		
latent profiles			
Low risk-high protection	0		
Medium risk-medium protection	0.91^***^	16.01	2.49 [1.59, 3.90]
High risk-low protection	2.44^***^	79.70	11.46 [6.71, 19.57]

**p < 0.01; ***p < 0.001.

## Discussion

There are intricate inducing mechanisms associated with NSSI in adolescents that should be explored from a broader and integrated perspective. In this study, LPA was employed on a larger sample to delve into a combined model of risk and protective factors. Based on model fit indices and theoretical understanding, the influences on adolescent NSSI were divided into three profiles: low risk-high protection, medium risk-medium protection and high risk-low protection. Adolescents classified into the medium risk-medium protection and high risk-low protection profiles exhibited a significantly greater likelihood of engaging in NSSI compared to those classified into low risk-high protection profile.

### Combined model of risk and protective factors for NSSI in adolescents

The low risk-high protection occupied 30.62% of the overall. This group showed the lowest levels of depression, and they tended to use more positive strategies for emotion regulation. They also reported high levels of regulatory emotional self-efficacy and resilience, and constructed a strong interpersonal protective network. Due to their reporting the lowest risk factors and the highest protective factor scores, this group typically exhibited higher levels of psychological health. Of the three profiles, the risk of NSSI for adolescents in the low risk-high protection was the lowest. This is consistent with previous studies that have taken a traditional variable-centered approach, examining only a single or few variables. Depression and expressive suppression, along with poor family functioning, were positively correlated with NSSI (23, 32, 48). Conversely, cognitive reappraisal, regulatory emotional self-efficacy, resilience, and social support were negatively associated with NSSI (23, 28, 42, 57).

More than half of the participants (58.20%) were classed as the medium risk-medium protection. Upon further analysis of this group’s characteristics, we found that poor family functioning was a prominent feature. Poor family functioning (52) was reported by adolescents who engage in NSSI. Additionally, their nearly equal use of cognitive reappraisal and expressive suppression may indicate confusion in emotion regulation strategies. Although they exhibited mild depressive symptoms, poorer family functioning, and confusing emotion regulation strategies, their regulatory emotional self-efficacy, resilience, and social support were higher. Notably, social support can play a pivotal role in enhancing emotion regulation and aiding in the more effective management of interpersonal stressors, such as those arising from family dynamics (e.g., conflicts or lack of communication) (77). Previous studies have also found that resilience and social support can mediate the effects of depression on NSSI (78, 79). This means they can regulate emotional and interpersonal distress through stronger resilience, emotional monitoring abilities, and seeking support. As a result, they are relatively less likely to experience serious psychological problems. Participants in the emotional distress group, as identified through LPA of suicide risk factors, were found to be susceptible to experiencing emotional distress, including anxiety, depression, and sleep disturbances. However, they demonstrated a greater capacity for coping through self-regulation and social support, resulting in a reduced likelihood of experiencing psychological abnormalities (80). This is similar to the medium risk-medium protection found in our study. Using binary logistic regression analyses, we discovered that adolescents classified as medium risk-medium protection exhibited over twice the likelihood, and those categorized as high risk-low protection had an elevated likelihood of over 11 times, to engage in NSSI compared to individuals with low risk-high protection profiles. Notably, despite the significant increase in risk among the high risk-low protection group, the relative risk increment appears modest when juxtaposed with the group displaying the highest vulnerability.

In the present study, only a small number of participants belonged to the high risk-low protection profile (11.18%). Individuals belonging to this category demonstrated the highest level of depression, which is likely attributable to significant deficiencies in their family functioning and inadequate social support. Previous studies have found family functioning and social support to be important predictors of depression (81, 82). They more frequently employed expression suppression, a negative emotion regulation strategy, to manage emotions. This finding aligns with previous research, which revealed that adolescents engaging in non-suicidal self-injury (NSSI) reported significantly higher scores on expression inhibition and correspondingly lower scores on cognitive reappraisal (24). Additionally, they reported the lowest self-efficacy for emotion regulation, indicating a lack of confidence and skills in managing emotions effectively. Finally, they reported the lowest levels of resilience. Previous research showed that cognitive reappraisal triggers adaptive emotional responses when individuals face adversity, which in turn significantly enhances their resilience (39). However, when individuals have low cognitive reappraisal scores, it means that they may lack the ability to effectively adjust their emotions, resulting in relatively weak resilience. In such situations, individuals may have difficulty recovering from adversity and may even be inclined to resort to NSSI behaviors as a way of relief (83). This group had a high prevalence of NSSI, with a likelihood of NSSI 11.46 times higher than that of the low risk-high protection profile. This result is also consistent with a cumulative effect, suggesting that the accumulation of diverse risk factors may increase an individual’s risk of NSSI. Therefore, it is crucial to pay special attention to and intervene with adolescents classified as the high risk-low protection profile. For adolescents in the high risk-low protection profile, targeted interventions should employ a multi-pronged strategy that simultaneously reduces risk factors (e.g., depression, emotion dysregulation) and enhances protective factors (e.g., resilience, social support). Evidence-based approaches to achieve this include cognitive-behavioral therapy (CBT) to address emotional difficulties, family systems therapy to improve family functioning, and school-based programs to strengthen social support networks, thereby helping them overcome challenges and reduce NSSI behaviors.

The integration model involves numerous factors and variables and provides a good theoretical model to guide the selection of detection factors. In reality, NSSI frequently arises from the concurrent and interacting influence of multiple risk factors, However, previous studies have primarily concentrated on the effect of one or a few risk factors on NSSI, leading to a decreased effectiveness and limited sensitivity in screening individuals for such behaviors. We selected seven representative predictors of NSSI based on the integrated model, which provided higher identification and differentiation capabilities compared to previous studies. However, validating the integration model from an empirical research perspective remains challenging due to the intricacy of the numerous factors and variables involved (Xu, 2014). With the development and application of LPA in recent years, the identification of NSSI risk based on the integration model has become possible. LPA offers numerous advantages in the identification of risk categories. It boasts more scientific and objective criteria in determining the number of profiles and classification characteristics, resulting in significantly improved classification accuracy compared to traditional methods. Additionally, LPA helps to identify heterogeneous subgroups of psychological behavioral problems and can effectively test the interactive effects between various factors. (84).

### Implications for future research

This study employed a cross-sectional research design and LPA to investigate the relationship between intra-individual and environmental factors and NSSI from an integrative perspective. However, it is challenging to draw causal inferences, and incorporating a longitudinal design alongside a causal experimental design in future research could enhance the generalizability of the findings. For instance, advanced statistical methodologies, including Latent Transition Analysis (LTA) and Random Intercept Latent Transition Analysis (RI-LTA), can be employed to delve profoundly into the risk-protection categorization of adolescents and their dynamic transition patterns across varying temporal junctures. Secondly, the generalizability of our findings may be limited by the characteristics of our sample, which consisted mainly of first-year university students and was predominantly female. Future studies should aim to include more diverse adolescent populations, such as middle school students, rural adolescents, and more balanced gender representations.

Thirdly, the study primarily relied on subjective reports from participants for the study variables, which may result in underestimation of risk factors or overestimation of protective factors due to social desirability (85). Future studies should consider collecting objective indicators to enhance the reliability of the results. Lastly, although gender and only-child status were controlled for, other potential confounders such as family income, trauma history, and peer influence were not considered. Furthermore, the limited selection of risk and protective factors may restrict the comprehensiveness of the findings. Future studies should include a broader range of covariates and factors to enhance the validity and precision of prevention and intervention strategies. For example, recent research has highlighted the role of mentalization—the capacity to understand one’s own and others’ mental states—in influencing emotion regulation and NSSI risk (86–88). Different types of mentalization may serve as either protective or risk factors depending on an adolescent’s emotional and interpersonal context (89). Although not measured in the current study, future research should consider incorporating mentalization as an important individual-level factor in the assessment of NSSI.

## Conclusion

Overall, the findings of the current study contribute to three profiles of risk and protective factors regarding NSSI, which enrich the existing literature. Compared to low risk-high protection, adolescents in medium risk-medium protection (OR = 2.49) and high risk-low protection (OR = 11.46) were significantly associated with increased odds of experiencing NSSI. The results offer valuable insights into the prevention of NSSI among adolescents. In particular, we should focus prevention efforts on individuals who exhibit high risk-low protection characteristics.

## Data Availability

The raw data supporting the conclusions of this article will be made available by the authors, without undue reservation.
